# Oral health and oral care in short‐term care: prevalence, related factors and coherence between older peoples’ and professionals’ assessments

**DOI:** 10.1111/scs.12667

**Published:** 2019-03-12

**Authors:** Susanne Koistinen, Lena Olai, Katri Ståhlnacke, Anna Fält, Anna Ehrenberg

**Affiliations:** ^1^ School of Education, Health and Social Studies Dalarna University Dalarna Sweden; ^2^ School of Medicine and Health School of Health Sciences Örebro University Örebro Sweden; ^3^ Department of Public Health and Caring Sciences Family Medicine and Preventive Medicine Uppsala University Uppsala Sweden; ^4^ Dental Research Department Postgraduate Dental Education Center Örebro Sweden; ^5^ Clinical Epidemiology and Biostatistics School of Medical Sciences Örebro University Örebro Sweden

**Keywords:** oral health, oral care, older people, short‐term care, self‐perceived, functional ability

## Abstract

**Background:**

Oral health is important for well‐being and overall health. Older peoples′ oral health is well described in the residential care context, but remains understudied in short‐term care.

**Objective:**

The aim of this study was to describe oral health, daily oral care and related factors among older people in short‐term care and to compare self‐perceived oral health with professional assessment.

**Materials and methods:**

This cross‐sectional study included 391 older people in 36 short‐term units in 19 Swedish municipalities. Oral health was assessed professionally by clinical oral assessment and the Revised Oral Assessment Guide (ROAG). The older peoples’ perceptions of their own oral health were measured with a global question on self‐perceived oral health. Self‐care ability was assessed with Katz Index of Activities of Daily Living (Katz‐ADL).

**Results:**

Mean age was 82.9 years, 19% of participants were totally edentulous, and 43% had ≥20 teeth. Almost 60% had coating or food debris on their teeth, but only 19% received help with daily oral care. Those who were dependent on help with self‐care had around a sixfold higher risk of having oral problems. There was a low level of agreement between the clinical assessment based on ROAG and self‐perceived oral health.

**Conclusion:**

Professionals’ assessments of oral health differed considerably from the older peoples′ own assessments. A higher risk of oral problems and more occurrence of coating or food debris or broken teeth were seen among those dependent on help with self‐care (ADL). This study indicates that in order to improve older peoples′ oral health and oral care we need to provide person‐centred oral care and to develop a close collaboration between nursing and dental staff.

## Introduction

The number and proportion of older people in Sweden is increasing, as in many other countries 1. Due to advances in oral health care and treatment in Europe 2, oral health among older people has improved in recent years, with fewer denture wearers and increasing numbers of natural teeth 3. Many old people have fixed constructions and implants instead of removable denture solutions 4, meaning that the combination of natural teeth and implants is becoming more common 5. It may be difficult to achieve good oral hygiene in a context including an increased number of natural teeth aided by restorative dentistry such as crowns, bridgework, partial dentures and implants 6. The conditions for maintaining good oral hygiene also become more challenging because part of the ageing process itself is a gradual decline in abilities such as sight and mobility 7. Remaining in good oral health requires adequate oral hygiene; otherwise, there is a risk of developing oral health problems 8. This may be especially challenging for older people who are dependent of help with their personal hygiene, as, for example, those who are cared for in short‐term care settings.

Good oral health is important for people's well‐being, nutrition, proper healing, self‐esteem, social satisfaction and quality of life as well as overall health 9, 10. The most prevalent oral diseases are caries and periodontitis, and good oral hygiene reduces the risk of their development 11. Poor oral health may threaten older people's general health and influence the initiation and/or progression of diseases such as myocardial infarction, stroke, diabetes, Alzheimer's disease and rheumatoid arthritis 12. Aspiration of bacteria can cause pneumonia and affect the development of chronic obstructive pulmonary disease in frail older people 12.

Although it can be assumed that clinical observations alone do not fully indicate how people experience their oral health, assessment of oral health is mostly performed by dental professionals and rarely takes the person's subjective perceptions and satisfaction with oral health into consideration 13. It is commonly observed that older people tend to have a more positive view of their oral health in comparison with professional assessments, even in situations where the clinical condition is assessed as poor 14. Self‐perceived oral health is often a combination of the history of an individual's behaviour, attitudes, culture, and experiences of their own oral health 15, 16. Many older people adapt to a deteriorating oral health, for example tooth loss, and view dental disease as a normal consequence of ageing 17.

Approximately 20% of the population in Sweden are 65 years or older. In 2016, there were almost 9% receiving home help services in their own home, about 4% lived in special housing, and the number of people in short‐term care was almost 1% 1. At a national level, about 80% of people in special housing, and almost 90% of those living with home support are satisfied with their care, according to people 65 years or older 18.

Swedish municipalities are responsible for health care in special accommodation and short‐term care for older people. Short‐term care is intended to meet the temporary care needs of older people following hospitalisation, awaiting a decision on permanent special accommodation or providing intermittent care, recurrent relief for family caregivers, rehabilitation and palliative care 19. The majority of older people come to short‐term care because of acute events such as stroke, fall injury or new diagnosis. About 90% of the people who receive short‐term care are living in ordinary housing, and the majority are aged 80–89 years 20. These older people have various conditions, and many are frail, with multiple disorders and diseases creating extensive care needs 19.

To obtain a holistic perspective on oral health and provide more person‐centred care, it is important to recognise people's perceptions of their own oral health and not just clinical indicators of oral disease 21. By working towards person‐centred care, the patient is actively involved in their care and decision‐making process. Person‐centred care has been shown to contribute to improved coherence between healthcare providers and patients on treatment plans, better health outcomes and increased patient satisfaction 22. One way of illustrating these perspectives is by applying Eriksson's ‘health cross’. Eriksson defined health as a two‐dimensional concept, illness and disease, where illness is the patient's self‐rated health and disease are their professionally assessed health 23.

There are several studies describing oral and dental health among older people living in special accommodation 8, 24, 25, 26; for example, in one study older people dependent on help with activities of daily living (ADL) had poorer oral health status and needed help with daily oral care 27. However, there is a lack of descriptions of the oral health of older people in short‐term care, as well as a lack of studies comparing older people's self‐perceived oral health and professionally assessed oral health. In order to develop person‐centred care, we need to take into account both the self‐perceived perspective and the clinical assessment of older people's oral health.

In conclusion, knowledge is limited about the oral health of older people and whether their oral care needs are met in short‐term care settings. The aim of this study was to describe oral health, daily oral care and related factors among older people in short‐term care and to compare the older people's self‐perceived oral health with professional assessment of oral health.

## Material and methods

### Design and setting

This descriptive cross‐sectional study was carried out within the framework of an ongoing research project conducted in five counties: SOFIA (Swallowing function, Oral health, and Food Intake in old Age). The overall aim of the SOFIA project is to describe and analyse oral health and oral health‐related quality of life, swallowing and eating ability, nutritional risk, care quality in relation to oral health and eating and to study the effectiveness of a swallowing training programme among older persons who are admitted to short‐term care 28. Thirty‐six short‐term units in 19 Swedish municipalities were selected by convenience based on their geographical location, number of beds and estimated numbers of discharges per month, and informed consent was achieved from the heads of social welfare services and unit managers. The municipalities represented both rural and urban areas 28 and were located in different parts of Sweden. Unit staff comprised nurse aides, licensed practical nurses, Registered Nurses, occupational therapists, physiotherapists and managers. Thus, the dental hygienist is not a part of the regular care team, but they offer oral health education to all nursing staff on annual basis 29. Healthcare staff are expected to provide oral care twice a day as part of routine daily care within elderly care.

### Participants

Older people admitted to the selected short‐term care units during a 3‐year period were eligible for study participation. The inclusion criteria were being 65 years or older, having spent at least 3 days at the short‐term care unit, being able to understand Swedish and having sufficient cognitive ability (judged by the nurse in charge) to answer questions 28. A population of 931 older people who were cared for in short‐term care were assessed for eligibility; of these, 477 (51%) did not meet the inclusion criteria. Reasons for exclusion were palliative care (n = 61), insufficient cognitive capacity (n = 309) or that the older persons had been admitted for <3 days, younger than 65 years or could not communicate in Swedish (n = 107). Of the 454 eligible participants, 63 (14%) declined to participate. A total of 391 older people were finally included in the study.

### Procedure

A convenience sample of five out of 21 Swedish counties was asked to participate. After approval by the head of social welfare of elderly care in each municipality, heads of unit were contacted to provide information on the study and request approval to visit the short‐term unit. The Registered Nurse in charge at the accommodation made an initial assessment about which older persons fulfilled the inclusion criteria and could be invited to participate in the study. The research assistants [eight registered dental hygienists (RDHs) and one speech language pathologist] informed the participants both orally and in writing about the purpose of the study and the procedures involved in participating, clarified the matter of confidentiality and obtained written consent. Questions about the older people's main medical diagnoses were answered by the Registered Nurse, and questions about the older people's self‐care ability were answered by the licensed practical nurse or the Registered Nurse. The RDHs carried out a clinical assessment using a mouth mirror and a flashlight and collected self‐reported questionnaire data by asking the participants questions 28. Each data collection lasted about 30–60 minutes. Data were collected from October 2013 to January 2016.

#### Ethical considerations

The data collection was conducted according to ethical principles and included informed consent, confidentiality and the right to withdraw from participation at any time without presenting a reason. If a severe oral health problem was detected, the research assistant informed the participant and the responsible nurse about the need to make contact with dental care for treatment. The study was approved by the Regional Ethical Review Board, Uppsala University, Sweden (Dnr 2013/100).

### Instruments

#### Assessment of functional status

Self‐care ability was assessed with Katz Index of Activities of Daily Living (Katz‐ADL) 30, 31, which summarises a person's overall performance concerning six functions: bathing, dressing and undressing, going to the toilet, mobilisation, controlling bowel and bladder, and food intake. Performance is graded from A to G, where A = independence in all functions, B = dependence on help in one activity, C = dependence on help in two activities, D = dependence in three activities, E = dependence in four activities, F = dependence in five activities and G = dependence in all respects 30. Katz‐ADL index is a widely used tool to assess the level of independency in older adults and it is tested for reliability and validity 31.

#### Clinical oral assessment

The oral assessment performed by the RDHs included recording the number of natural teeth, presence of bridges, partial or full dentures and implants, need for dental care and an estimation of oral hygiene in terms of three categories from good to poor. Additionally, one question was asked about the person's ability to brush their own teeth, with three response options: 1 = ‘Yes, completely able’, 2 = ‘Receive some help’ and 3 = ‘No, receive help entirely’ 28.

#### Revised Oral Assessment Guide (ROAG)

Oral health was measured using an adapted version of ROAG: the Revised Oral Assessment Guide‐Jönköping (ROAG‐J) 32. ROAG is a systematic assessment tool designed for use by nursing staff to detect problems related to mouth, teeth and dentures in older people 24, 33. Nine categories are included voice, lips, mucous membranes, tongue, gums, teeth, dentures, saliva and swallowing 32. Each category is graded on a three‐point scale where 1 = ‘healthy’, 2 = ‘moderate oral health problem’ and 3 = ‘severe oral health problem’ 24, 33.

#### Self‐perceived oral health

A global question was used to assess self‐perceived oral health: ‘Are you generally pleased with your mouth and your teeth?’. There were four response alternatives, ranging from ‘very satisfied’ to ‘not at all satisfied’ 34.

### Statistical analyses

Descriptive results are shown as frequencies with percentages or means with standard deviations (SD). The ADL index was divided into three categories: A = independent, B–D = partly dependent and E–G = completely dependent 31, 35. Self‐perceived oral health was dichotomised as 0 = ‘Very satisfied’ or ‘Largely satisfied’ and 1 = ‘Not very satisfied’ or ‘Not at all satisfied’. In the regression and agreement analyses, the ‘teeth’ and ‘dentures’ items in ROAG were merged into a single item, giving eight items with a total score ranging from 8 (healthy) to 24 (severe oral health problems). The total score was then dichotomised as 0 = no oral problems (score 8) and 1 = oral problems (score 9–24) 27.

Percentage agreement and Cohen's kappa coefficient (k) were calculated to measure the agreement between the clinical oral assessment (ROAG: no oral problems vs. oral problems) and the older people's self‐perceived oral health (satisfied vs. not satisfied). Overall percentage agreement was calculated by taking the number of agreements between the two measurements, dividing this by the total number of readings and multiplying the result by 100. Agreement was considered to occur when either both the older person and the RDH assessed oral health as good (i.e. ‘satisfied with oral health’ and ‘no oral problems’) or when both assessed oral health as poor (i.e. ‘not satisfied with oral health’ and ‘oral problems’). Cohen's kappa coefficient adjusts for agreements due to chance, and values <0.2 are considered as poor, 0.21–0.40 as fair, 0.41–60 as moderate, 0.61–0.80 as good and >0.80 as very good agreement 36, 37.

Two separate multivariate logistic regression analyses were conducted, yielding adjusted odds ratios (ORs) with 95% confidence intervals (CIs). The dependent variable in the first analysis was self‐perceived oral health (0 = satisfied; 1 = not satisfied), and the dependent variable in the second analysis was oral problems based on clinical assessment (ROAG; 0 = no oral problems, 1 = oral problems). The independent variables in both analyses were gender, age, education, number of teeth, removable dentures, oral self‐care, need for dental care and ADL index. Statistical significance was set at p < 0.05. Data were analysed using version 22 of the ibm spss software package (IBM, Armonk, NY, USA).

## Results

The results are based on 391 older people from 36 short‐term care units in five counties. Their ages ranged from 65 years to 100 years (m = 82.9, SD = 7.7), and they comprised 209 (53%) women and 182 (47%) men (Table 1). Statistical differences were between men and women regarding age, education and dependency on help with activities of daily living. Their main medical diagnoses were stroke (n = 87, 22%), musculoskeletal disease/locomotor disorder (n = 85, 22%) and mild cognitive impairment (n = 47, 12%), and 206 (53%) of them had three or more medical diagnoses. The most common reasons for admission were respite care (n = 76, 19%), acute short‐term care (n = 70, 18%), recovery after hospitalisation (n = 58, 15%), rehabilitation (n = 50, 13%) and awaiting arrangements for permanent housing (n = 33, 8%).

**Table 1:** Participant characteristics

	Men n = 182 (%)	Women n = 209 (%)	Total n = 391 (%)
Age, years			
65–84	113 (62)	99 (47)	214 (54)
85–100	69 (38)	110 (53)	179 (46)
Education (n = 386)			
Compulsory school	104 (58)	147 (71)	251 (65)
Upper secondary school	56 (31)	43 (21)	99 (26)
University	20 (11)	16 (8)	36 (9)
Katz′s ADL index (n = 358)			
A	14 (8)	14 (7)	28 (7)
B–D	61 (34)	104 (50)	165 (43)
E–G	104 (58)	88 (43)	192 (50)

### Assessment of oral health

A total of 74 (19%) older people were completely edentulous, and 167 (43%) had 20 teeth or more. The presence of removable dentures (full or partly), implants and bridges was indicated if they were observed either in one jaw or both jaws. Two people had missing data regarding dental status (Table 2). In terms of oral hygiene, 164 (46%) of the older people were assessed as having good oral hygiene and 190 (54%) had less good to poor oral hygiene. A total of 148 (41%) were assessed to have a need for dental care treatment. Finally, 310 (79%) performed oral self‐care independently, 56 (14%) received some help, and 18 (5%) received help entirely; data were missing for the remaining 7 (2%). There were no statistical significant differences in dental status for gender or for age.

**Table 2:** Dental status among older people (n = 389) based on clinical assessment by dental hygienists

Dental variables	N (%)
Number of teeth	
Edentulous	74 (19)
Teeth 1–19	148 (38)
20–32	167 (43)
Removable dentures (full, partly)	
Yes	135 (35)
No	254 (65)
Implants	
Yes	33 (9)
No	356 (91)
Bridges	
Yes	133 (34)
No	256 (66)

### Oral health based on ROAG

The most frequent oral health problem was in the teeth category, specifically the presence of coating or food debris, which was seen in 183 (57.2%) of the older people. Older people with implants assessed having coating or food debris are graded in category teeth. Numbers and percentages of identified oral health problems are shown in Table 3.

**Table 3:** Oral health in terms of the Revised Oral Assessment Guide among older people (n = 390) in short‐term care based on clinical assessment by dental hygienists

Item Category	Grade 1 Findings N (%)	Grade 2 N (%)	Grade 3 N (%)
Voice	Normal 252 (65.1)	Dry, hoarse, smacking 112 (28.9)	Difficult to speak 23 (6.0)
Lips	Smooth; bright red; Moist 322 (83.4)	Dry, cracked, sore corners of the mouth 62 (16.1)	Ulcerated, bleeding 2 (0.5)
Mucous membranes	Bright red; moist 325 (85.8)	Red; dry or areas of discoloration, coating 52 (13.7)	Wounds, with or without bleeding, blisters 2 (0.5)
Tongue	Pink, moist with papillae 303 (78.7)	No papillae, red, dry coating 79 (20.5)	Ulcers with or without bleeding, blistering 3 (0.8)
Gums	Light red and solid 243 (71.1)	Swollen, reddened 93 (27.2)	Spontaneous bleeding 6 (1.7)
Teeth	Clean; no visible coating, food debris 137 (42.8)	Coating or food debris locally 146 (45.6)	Coating, food debris generally or broken teeth 37 (11.6)
Dentures	Clean; works 53 (39.0)	Coating or food debris 77 (56.6)	Not used or malfunctioning 6 (4.4)
Saliva	Glides easily 304 (78.4)	Glides sluggishly 78 (20.1)	Does not glide at all 6 (1.5)
Swallow	Unimpeded swallowing 287 (76.3)	Insignificant swallowing problems 66 (17.6)	Pronounced swallowing problems 23 (6.1)

Score on Katz's ADL index was associated with the ability to brush one's own teeth (p < 0.001). None (0%) of the older people with grade A (total independence) received either partial or total help, while the corresponding figures for grades B–D (dependence in one to three ADL) and E–G (dependence in four to all six ADL) were 12 (7.3%) and 61 (33%), respectively. ADL index was also associated with dental status according to ROAG (p = 0.002). Seven (33%) of the older people with grade A had local/general coating or food debris or broken teeth, while the corresponding figures for grades B–D and E–G were 70 (52%) and 105 (67%), respectively.

### Self‐perceived oral health and clinical assessment of oral health based on ROAG

A majority of the older people (n = 321, 85%) reported being very satisfied or generally satisfied with their oral health. However, the assessment based on ROAG found oral problems in 297 (77%) of the total group.

**Figure 1:**
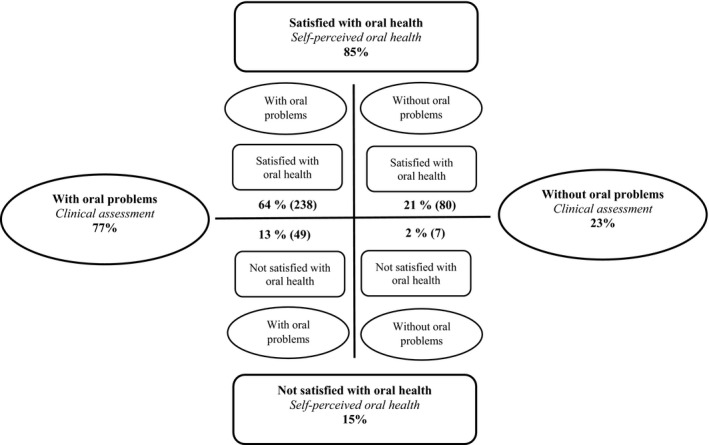
Percentage agreements between clinical assessment (ROAG) and the older people's self‐perceived oral health (n = 374). The figure is based on the ‘health cross’ described by Eriksson 23.

When comparisons were made between the assessment based on ROAG and the older people's self‐perceived oral health, a low level of agreement was found. The kappa coefficient showed very poor agreement (*k* = 0.047), and the overall percentage agreement between professional assessment and the older people's self‐perceived oral health was only 34%. Overall, 21% of the older people were both satisfied with their oral health and clinically assessed as being without oral problems. Oral health was assessed by RDHs as being worse than the participants’ perceptions in 64% of all assessments and better than the participants’ perceptions in 2% of the assessments. The percentage agreements between professional assessment and the older people's self‐perceived oral health are presented in Fig. 1.

### Associations between different factors and the older people's self‐perceived oral health and oral health based on ROAG

Table 4 presents the adjusted ORs for dissatisfaction with oral health (model 1) and having oral problems based on clinical assessment using ROAG (model 2). Participants with university or higher education had 4.7 times higher odds for dissatisfaction with oral health compared to participants with only compulsory education (OR: 4.69; 95% CI: 1.58–13.95). Participants with an observed need of dental care were eight times more likely to be dissatisfied with oral health compared to participants with no such need (OR: 8.38; 95% CI: 3.81–18.43).

**Table 4:** Adjusted odds ratios (ORs) with 95% confidence intervals (CIs) for dissatisfaction with oral health (model 1) and oral problems based on clinical assessment using the Revised Oral Assessment Guide (model 2), in relation to various demographic and clinical characteristics

	Adjusted model 1 OR (95% CI)	p	Adjusted model 2 OR (95% CI)	p
Gender				
Female	1.00 (ref)		1.00 (ref)	
Male	1.03 (0.51–2.08)	0.930	1.24 (0.71–2.18)	0.456
Age (cont.)	1.01 (0.96–1.06)	0.713	0.99 (0.96–1.04)	0.878
Education				
Compulsory school	1.00 (ref)		1.00 (ref)	
Upper secondary school	1.01 (0.45–2.29)	0.979	0.58 (0.31–1.08)	0.085
University	4.69 (1.58–13.95)	0.005	0.79 (0.33–1.92)	0.606
Number of teeth				
0 teeth	1.00 (ref)		1.00 (ref)	
1–19 teeth	2.71 (0.97–7.62)	0.058	0.60 (0.24–1.51)	0.277
20–32 teeth	0.48 (0.13–1.82)	0.280	0.32 (0.11–0.98)	0.045
Removable dentures (full, partly)				
No	1.00 (ref)		1.00 (ref)	
Yes	1.64 (0.72–3.70)	0.238	0.69 (0.28–1.68)	0.409
Perform oral self–care				
Yes, completely	1.00 (ref)		1.00 (ref)	
Receive some help	1.21 (0.44–3.31)	0.708	2.03 (0.77–5.36)	0.151
No, receive help entirely	0.29 (0.03–2.86)	0.286	1.78 (0.36–8.86)	0.484
Need for dental care				
No	1.00 (ref)		1.00 (ref)	
Yes	8.38 (3.81–18.43)	<0.001	4.74 (2.41–9.34)	<0.001
Katz′s ADL index				
A	1.00 (ref)		1.00 (ref)	
B–D	0.61 (0.17–2.14)	0.439	2.30 (0.89–5.95)	0.085
E–G	0.55 (0.15–1.99)	0.364	3.36 (1.27–8.92)	0.015
Nagelkerke's pseudo–*R* ^2^	0.324			0.205

Furthermore, participants with 20–32 teeth were 70% less likely to have oral problems (based on clinical assessment) compared to participants with no teeth (OR: 0.32; 95% CI: 0.11–0.98). The odds for having oral problems were nearly five times higher for participants with a need for dental care compared to those with no such need (OR: 4.74; 95% CI: 2.41–9.34). Finally, participants with ADL index E‐G (dependence in four to all six ADL) had 3.4 times higher odds of having oral problems compared to participants who were independent (ADL index A) in all activities (OR: 3.36; 95% CI: 1.27–8.92). There were no other statistically significant results.

## Discussion

This study shows that professional assessments of oral health differed considerably from the self‐perceived oral health of older people in short‐term care settings. Although the majority of the older people had oral health problems, only 19% received help with daily oral care. Older people who were dependent on help with self‐care according to Katz's ADL index had around a sixfold higher risk of oral problems and a doubled risk for presence of local/general coating or food debris or broken teeth, according to ROAG.

To our knowledge, this is the first study comparing self‐perceived oral health and clinical assessment among frail older people in the context of short‐term care. A large majority (85%) of the older people reported themselves as being very satisfied or generally satisfied with their oral health, while the RDHs only identified 23% to be without oral problems. The older people in this study had a high number of remaining natural teeth, which confirms findings from recent studies in Sweden 3 and other European countries 2. The fact that older people retain more own natural teeth, compared to previous generations, with high number of people with dentures or edentulous, might contribute to older peoples good perception of their oral health. This discrepancy points to the importance of both asking older people about their self‐perceived oral health and making professional assessment in order to provide a more person‐centred care. According to Cohen's kappa, the strength of agreement was very poor (*k* = 0.047). These findings are in agreement with a previous study which showed that older people's self‐perceived oral health often differed from health professionals’ oral assessments, with nursing staff assessing oral health as being poorer than the patients did 38. In health care, differences have been shown between nursing staff's objective assessment of the patient and the patient's subjective experience 39. Other studies report that many older people have good self‐perceived oral health 9, 40. Explanations for older people's more positive perceptions of their oral health are often a combination of the history of an individual's behaviour, attitudes, culture and experiences of their own oral health 15, 16. In addition, older people can adapt to, for example, tooth loss and view dental disease as a normal consequence of ageing 17. We used Eriksson's ‘health cross’ to illustrate the coherence between clinical assessment and the older people's self‐perceived oral health 23. One dimension shows the presence or absence of objective illness, and the other dimension shows the individual's experience of themselves as ill or healthy 23. It is important to recognise both these dimensions in order to obtain a holistic perspective of oral health and provide person‐centred care 21.

Almost half of the older people were assessed as having less good to poor oral hygiene, and according to the ROAG assessment, coating or food debris was present locally or generally on both teeth and dentures in almost 60% of the older persons. This indicates a lack of proper oral care and points to the importance of regular oral assessments. Despite the observed care needs, only a fifth of the older persons received any help from care staff with their daily oral care. This low level of help with oral care seems inadequate, considering the large number of older people in the sample who were highly dependent on help with activities of daily living. According to the ADL assessment, half of the sample were dependent on help with four to six activities such as bathing, dressing and undressing, going to the toilet, mobilisation, controlling bowel and bladder, and food intake. Only about one‐third of the older people in this group received partial or total help with oral self‐care. A study from a geriatric ward in Sweden found that patients depending on help with ADL also had poor oral health status, which indicates that older people who depend on support with personal hygiene should also be assumed to need help with oral self‐care 27. A recent study from South Korea among older people living in long‐term care facilities also found that ADL was a significant predictor of oral hygiene 41.

The combination of natural teeth, removable dentures and bridges also leads to more complex oral care needs 42. This in turn makes oral care even more demanding to perform for nursing staff with limited education in oral care. Previous studies have shown that barriers for nursing staff in assisting with oral care often involve the older person resisting oral care 43, 44, 45, and the provision of such care can be experienced as an intrusion into the older people's personal integrity 46. Lack of time and sometimes other work tasks that are given higher priority can also be barriers to attending to oral care needs, as oral care is perceived as quite time consuming 44. Some nursing staff consider oral care an unpleasant task 43, 44, and some find it difficult because they themselves suffer from dental fear 44. Other barriers to assisting older people with oral care include lack of knowledge, education or training in providing oral care among nursing staff 45. There seems to be a separation between oral care and other nursing activities, as oral care is not discussed during nursing planning but only when oral problems arise 47. Oral care should be an activity central to caring and seen as equally important as other ADL when caring for older people with decreased self‐care ability 48. In order to improve oral hygiene status among older people, nursing staff need increased motivation for daily oral care tasks 43. Many older people in short‐term care are frail with multiple disorders, diseases and complex healthcare needs 19, and so it is of great importance that they remain in good oral health to maintain their social well‐being, nutrition, overall health and quality of life 9, 10, 38.

Interventions to improve oral care among older people in special accommodation should include both nursing staff and dental care staff to foster teamwork 49. Such teamwork may also enhance development of new knowledge and work procedures. A recent study shows that individual hands‐on‐guidance on a regular basis by dental hygienist to both older people and nursing staff in nursing homes improved oral health among older people 50. It is important that all staff involved in care of older people have basic knowledge of oral health and oral care 51.

Factors such as gender, age, number of teeth, removable dentures, ability to perform oral self‐care and dependence on help with ADL did not influence the older people's self‐perceived oral health in this study. However, older people who were not satisfied with their oral health had higher educational level and higher need of dental care. We also examined which factors may affect older people's oral problems based on clinical assessment with ROAG. Older people who were dependent on help with self‐care (in one up to all six activities according to Katz's ADL index) had around a sixfold higher risk of oral problems. This result is in line with previous studies that also found increasing dependency to be associated with oral health problems among older people in need of care 27, 52. Further efforts are needed to ensure that older people with high dependence in daily activities also receive help with oral care as an integrated part of their daily care. Older people's ability to perform oral self‐care should be included in the assessment of people's self‐care ability 41. These results can be used in the education of nursing staff to influence changes in daily oral care. It can also be a first step to improve daily oral care by influencing policy and practice when the study findings are reported on community level.

An experience gained during the data collection, as well as recognised from daily practice, is that some older people are reluctant to accept assistance with their oral care, although this is offered. The reason for the imbalance between oral care needs and care provided should to be further studied. Qualitative studies should be conducted to find out why older people do not always receive, or accept, help with oral care and to explore their experiences of receiving oral care. It is also important to increase our knowledge about the different factors that impact older people's self‐perceived oral health and oral health‐related quality of life.

These results can be useful for both dental and nursing staff to improve older peoples′ oral health, by ensuring good daily oral care. It also shows that it is not enough to just ask an older person about oral health, an assessment of the oral health also needs to be performed. Oral health and the ability to independently perform daily oral care should be examined in a similar way as ADL capacity.

### Methodological considerations

There is a lack of studies conducted in the short‐term care context, which may be due to methodological and ethical problems related to obtaining informed consent and controlling for confounding factors. It is challenging to include older people with weak health and functional disabilities 53. The results of this study are not fully representative of the population studied (older people in short‐term care), since it is based on a convenience sample of units and over 50% of those eligible did not meet the inclusion criteria. The fact that 477 of the eligible persons were excluded in the study shows that many older people in short‐term care are too frail to be eligible to participate. Nevertheless, it is important to involve this group of older people in research as they have high level of dependence and may be affected by flaws in basic care. It is reasonable to believe that those older people who were excluded had more severe oral health problems and a worse self‐perceived oral health than the sample, which might have affected the results.

The research assistants read the questions to the participants in order to make it easier to understand and answer all the questions. Answering questionnaires might be exhausting for older people, and misunderstandings could be corrected by supporting the participants in completing the task. On the other hand, there might be a risk of bias if older people are given support in reading and interpreting the questions. The strength of the assessments was that the research assistants who collected data on oral health were registered dental hygienists with relevant clinical experience in communicating with older people and assessing their oral health. The examinations were either conducted in the morning, after lunch or in the afternoon and sometimes divided over days, dependent on the older person's ability to participate. This might reduce the risk of bias as the timing in relation to food intake and oral care and oral examinations varied. All research assistants were trained in using the different instruments and met regularly with the research group to ensure consistency in assessments. The duration of the study was approximately two and a half years due to the inclusion of one more county in order to achieve sufficient power for the larger SOFIA study, which this study was part of 28. Because the short‐term units were selected by convenience, generalisability of the result should be made with some caution. Since legislation and regulations in Sweden provide some uniformity in staffing and quality of care, there could be smaller variations in contextual factors in the included municipalities and units. Data were collected from 36 different short‐term care units located in both rural and urban areas in five different counties in Sweden, which improves the generalisability of the findings.

## Conclusions

There was poor agreement between professional clinical assessment of oral health and self‐perceived oral health among older people in short‐term care. The majority of the participants was satisfied with their oral health although the clinical assessment often showed poor oral health. Those dependent on help with self‐care (ADL) had much higher risk of having oral problems and more occurrence of coating or food debris or broken teeth. This all together demonstrates the importance of providing person‐centred oral care and that close collaboration between nursing and dental staff must increase in order to improve older peoples′ oral health and oral care.

## Author contributions

SK designed the study, collected data, analysed data and drafted the paper. AF contributed with statistical analyses and draft of the paper. LO, KS and AE contributed in designing, analysing and drafting of the paper.

## Ethical approval

Ethical approval was obtained from the Regional Ethical Review Board, Uppsala University, Sweden (Dnr 2013/100).

## Funding

This study was supported by grants from Örebro County; the Regional Research Board of Uppsala‐Örebro; the Swedish Research Council for Health, Working Life, and Welfare (Forte); the Kamprad Family Foundation for Entrepreneurship, Research, and Charity. This study was accomplished within the context of the Swedish National Graduate School for Competitive Science on Ageing and Health (SWEAH), funded by the Swedish Research Council.
